# Paraganglioma of Urinary Bladder in a Pediatric Patient

**DOI:** 10.7759/cureus.13964

**Published:** 2021-03-18

**Authors:** Shoaib Muhammad, Amman Yousaf, Arif Qayyum, Rabia Nazim, Muhammad Taqi

**Affiliations:** 1 Department of Urology, Ghulab Devi Hospital, Al-Aleem Medical College, Lahore, PAK; 2 Department of Radiology, Hamad General Hospital, Doha, QAT; 3 Department of Radiology, Services Hospital Lahore, Lahore, PAK; 4 Department of Community Medicine, King Edward Medical University, Lahore, PAK; 5 Department of Anaesthesiology, Ghulab Devi Hospital, Al-Aleem Medical College, Lahore, PAK

**Keywords:** extra-adrenal paraganglioma, urinary bladder ca, pediatric

## Abstract

Paragangliomas arise from neural cells and are found in different anatomical locations in the body. Paragangliomas in the adrenal glands are called pheochromocytoma, while the others are known as extra-adrenal paragangliomas. They are usually benign and are extremely rare in children. We present a case of a 13-year-old female patient who presented with complaints of hematuria for one year and left lower lumbar pain. Imaging investigations depicted a urinary bladder mass that was causing a mass effect at the left ureteric opening and backpressure changes in the left kidney. The patient underwent transurethral resection of bladder mass, and the histopathology confirmed the presence of paraganglioma. Though the paragangliomas of the urinary bladder are extremely rare in the pediatric age group, we suggest keeping paragangliomas on differentials when investigating a patient with bladder mass.

## Introduction

Pheochromocytoma and paraganglioma are neural cell origin tumors [1]. Being histologically the same, they are differentiated based on their anatomic location, with pheochromocytoma being an adrenal gland tumor and paraganglioma being an extra-adrenal tumor. Compared to pheochromocytoma (80-85%), paragangliomas are extremely uncommon (15-20%) [1]. These tumors are usually diagnosed at the ages of 30 to 50 years, and diagnosis in children is extremely rare (10%). Most tumors are benign; however, infiltration and metastasis go in the favor of malignancy [2].

Paragangliomas in the urinary bladder being a rare entity has a prevalence of around 0.06% of all tumors, and those with malignant potential advocated by metastasis are extremely rare [3,4]. The first case of paraganglioma of the urinary bladder was reported in 1953, and since then only a few hundred cases have been reported [4]. We present a case of urinary bladder paraganglioma in a pediatric age group patient who was diagnosed during the workup for hypertension and lumbar pain. We suggest keeping paraganglioma in the differential diagnosis of the urinary bladder mass especially those who present with hypertension.

## Case presentation

A 13-year-old girl presented to the emergency department with a history of hematuria for the last one year associated with on and off left lumbar pain. Her family history was insignificant for any malignancy of the genitourinary tract. Her pulse was 94 beats/minute, blood pressure was 150/90 mmHg, and respiratory rate was 17 breaths/minute. Here general physical and abdominal examination was unremarkable.

**Figure 1:**
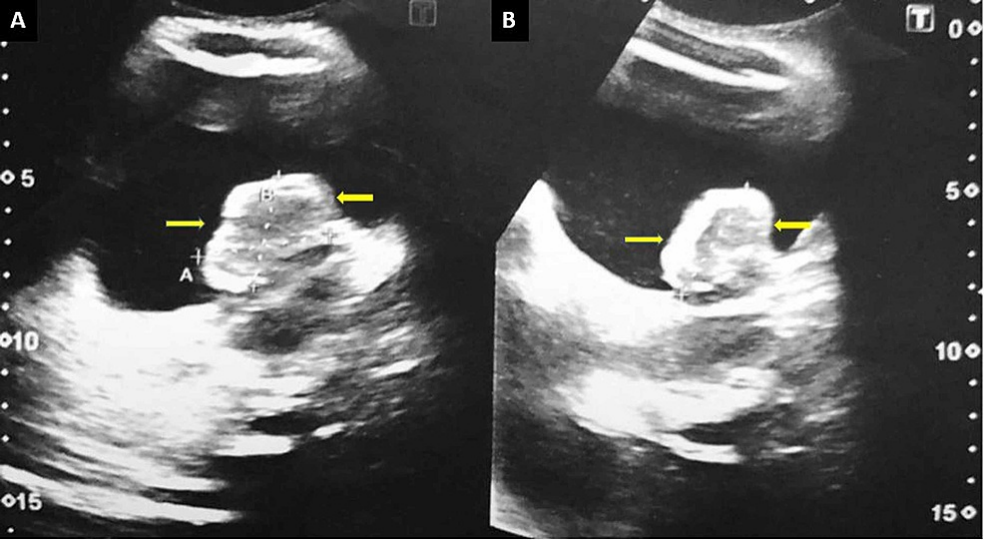
Ultrasound of the urinary bladder showing a large lobulated heterogeneously hypoechoic mass originating from the posterior wall.

Ultrasound (USG) of kidneys and urinary bladder was performed with a full bladder, and an irregular mass was appreciated in the left posterior wall of the urinary bladder with significant left-sided hydronephrosis (Figure 1).

**Figure 2:**
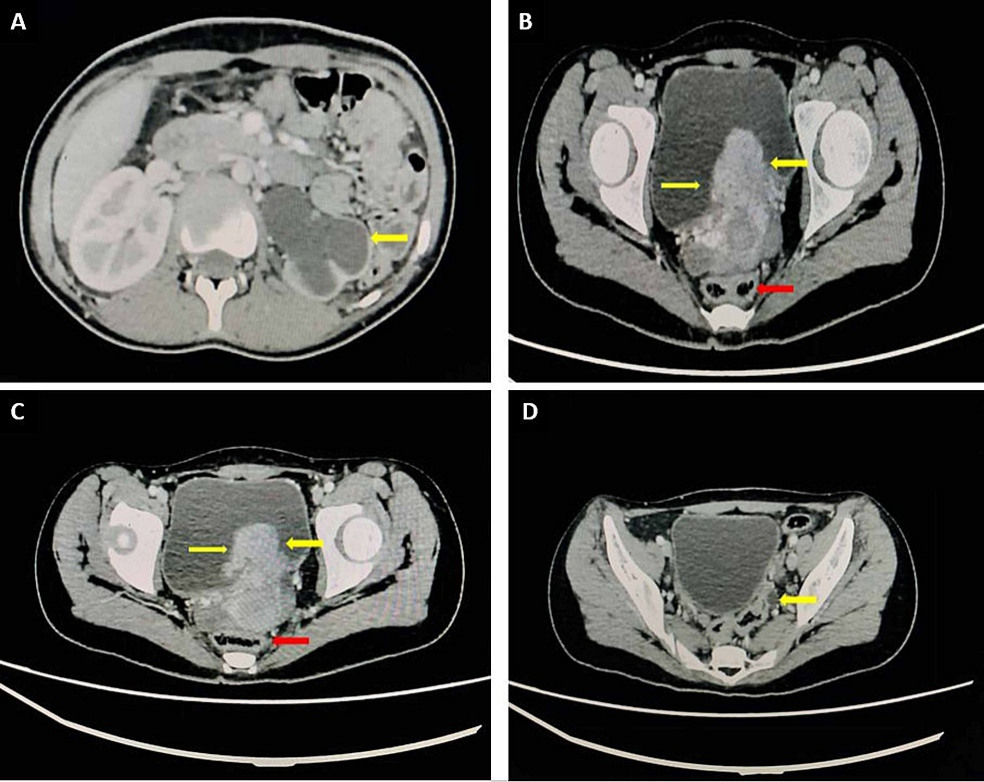
Contrast-enhanced computed tomography (CT) of the abdomen and pelvis (A) Axial selected section at the kidneys level, demonstrating left-sided dilated pelvicalyceal system with thinning of the cortex (hydronephrosis). (B & C) Selected axial images demonstrating a large heterogeneously enhancing mass lesion (yellow arrows) arising from the left posterior wall of the urinary bladder with no obvious rectal (red arrows) infiltration. (D) Dilated distal left ureter (arrow).

Contrast-enhanced computed tomography (CT) confirmed a large urinary bladder mass in the left margin of the urinary bladder base. It had heterogeneously enhancing soft tissue density and was abutting the anterior perirectal wall and the adjacent structures. Multiple enlarged lymph nodes were seen in the left pelvic area (Figure 2).

**Figure 3:**
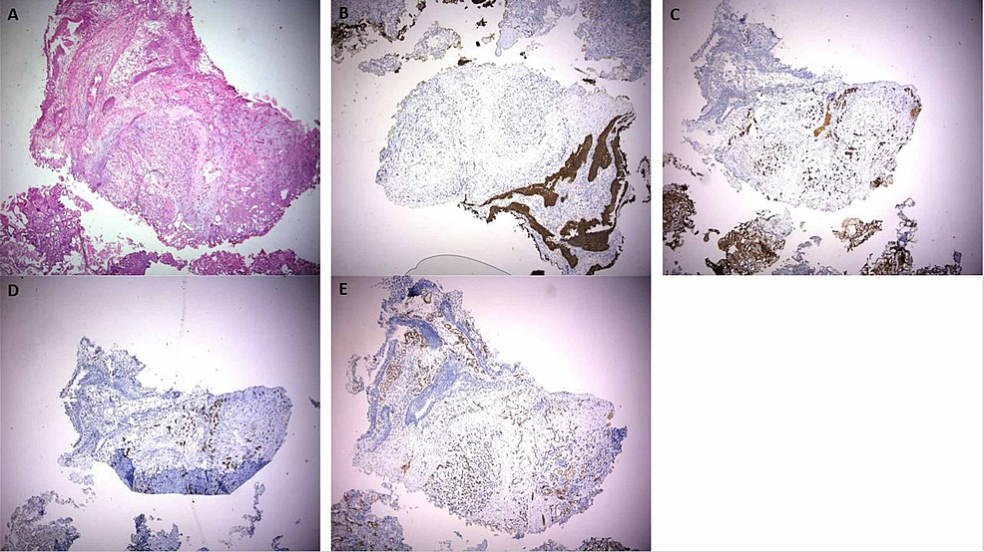
Histopathological features of the resected urinary bladder mass Tumor cells in the urinary bladder mucosa arranged in nests and trabeculae. Individual cells are round to oval with eosinophilic cytoplasm and atypical nuclei (A). Tumor cells are negative for cytokeratin (B), whereas they are positive for chromogranin (C), synaptophysin (D), and S-100 (E).

The multidisciplinary team decided to proceed with transurethral cystoscopic resection of the mass with special preoperative and intra-operative preparation (phenoxybenzamine and propranolol) to prevent any hypertensive crisis. Surgery and postoperative recovery went uneventful. Histopathology of the resected specimen revealed a focus of tumor cells within the bladder mucosa. The tumor cells were arranged in nests and trabeculae. Individual cells were round to oval with abundant cytoplasm and atypical nuclei. The immunohistochemistry was significant for chromogranin, synaptophysin, and S100 positivity (Figure 3).

A diagnosis of urinary bladder paraganglioma was made. The patient was discharged on the third postoperative day and is living a routine healthy life to date.

## Discussion

Paragangliomas originating in the urinary bladder are extremely uncommon tumors that grow from chromaffin tissue of the sympathetic nervous system related to the urinary bladder. These growths can be active (producing secretions) or inactive (not producing any secretions) [3]. The paragangliomas can present with the clinical picture of excessive production of catecholamine (hypertension, tremors, diaphoresis, headache, and loss of consciousness after passing urine). The first case of urinary bladder paragangliomas was reported by Zimmerman et al. in 1953 [4].

Paragangliomas occurring in the urinary bladder make 0.05% of all tumors. In the genitourinary system, the urinary bladder is the most frequent site for the occurrence of paragangliomas (79.2%), and the second most common site is the urethra (12.7%) followed by the pelvis (4.9%) and ureter (3.2%). Female predominance has been reported in the literature with a female-to-male ratio of 3:1, and they usually present between 30 and 50 years of age [5,6]. Most tumors occurring outside adrenal glands are found in the abdomen. Around 85% of them present under the diaphragm on the side of the sympathetic chain on the site of aortic division or the beginning of the inferior mesenteric artery (from the organ of Zuckerkandl) [7]. The least frequent site of occurrence is the urinary bladder [8].

Due to the absence of typical features, these are sometimes misdiagnosed as urothelial cancers. Around 10% of the paragangliomas in the urinary bladder are malignant [4]. Gross or histological features are not enough to differentiate between benign and malignant paragangliomas. Local infiltration of the surrounding organs, lymph nodes involvement, and metastasis to the remote organs are the characteristic features of malignant paragangliomas [8-10].

The paragangliomas of the urinary bladder usually have a lobulated macroscopic appearance. On microscopy, the tumor cells are arranged in trabeculae and nests making the zellballen pattern. Cells have acidophilic granular cytoplasm with oval-shaped nuclei [10]. Although paragangliomas occur in various locations, they usually show almost similar imaging features. CT demonstrates the paraganglioma as a heterogeneous soft tissue mass with a homogeneous or heterogeneous contrast enhancement followed by slow washout owing to a high capillary network [11]. These features were consistent with the findings in our case. On MRI, the lesions tend to be hypointense or isointense on T1 and hyperintense on T2 with interspersed areas of signal voids (salt-and-pepper appearance), with heterogeneous contrast enhancement. Metaiodobenzylguanidine scintigram (MIBG) has a crucial role in differentiating between functional and non-functional paragangliomas [10]. Surgical resection is the treatment of choice with adequate pre- and postoperative preparation to avoid the hypertensive crisis. Radical cystectomy, partial cystectomy, and transurethral resection of the tumor are the available surgical options [10,12-14]. In our case, transurethral resection was performed as the tumor was a single focus, with no infiltration of the surrounding structures and no distal metastasis.

## Conclusions

Paragangliomas can occur in a variety of anatomical locations.. The imaging features of paragangliomas of different locations are almost identical, and local or distal metastasis differentiates between benign and malignant lesions. We suggest keeping paraganglioma in the differentials of a solid urinary bladder mass lesion in pediatric age group patients.
